# MFSD2A is a novel lung tumor suppressor gene modulating cell cycle and matrix attachment

**DOI:** 10.1186/1476-4598-9-62

**Published:** 2010-03-17

**Authors:** Monica Spinola, Felicia S Falvella, Francesca Colombo, James P Sullivan, David S Shames, Luc Girard, Paola Spessotto, John D Minna, Tommaso A Dragani

**Affiliations:** 1 Department of Predictive and for Prevention Medicine, Fondazione IRCCS Istituto Nazionale Tumori, Milan, Italy; 2 Hamon Center for Therapeutic Oncology Research, University of Texas Southwestern Medical Center, Dallas, TX, USA; 3 Simmons Comprehensive Cancer Center, University of Texas Southwestern Medical Center, Dallas, TX, USA; 4 Divisione di Oncologia Sperimentale 2, Centro di Riferimento Oncologico di Aviano, Aviano, Italy

## Abstract

**Background:**

MFSD2A (major facilitator superfamily domain containing 2) gene maps on chromosome 1p34 within a linkage disequilibrium block containing genetic elements associated with progression of lung cancer.

**Results:**

Here we show that MFSD2A expression is strongly downregulated in non-small cell lung cancer cell lines of different histotypes and in primary lung adenocarcinomas. Experimental modulation of MFSD2A in lung cancer cells is associated with alteration of mRNA levels of genes involved in cell cycle control and interaction with the extracellular matrix. Exogenous expression of MFSD2A in lung cancer cells induced a G1 block, impaired adhesion and migration *in vitro*, and significantly reduced tumor colony number *in vitro *(4- to 27-fold, P < 0.0001) and tumor volume *in vivo *(~3-fold, P < 0.0001). siRNA knockdown studies in normal human bronchial epithelial cells confirmed the role of MFSD2A in G1 regulation.

**Conclusion:**

Together these data suggest that MFSD2A is a novel lung cancer tumor suppressor gene that regulates cell cycle progression and matrix attachment.

## Background

Cancer progression is defined as the stepwise process through which cells evolve towards a more malignant and aggressive phenotype [1]. This process results from the accumulation of somatic genetic and epigenetic changes occurring within neoplastic cells [2]. However, a growing body of evidence also points to the role of genetic background in cancer susceptibility, progression, and prognosis [3-5]. We previously identified a 106 kb linkage disequilibrium block containing genetic elements associated with survival in lung adenocarcinoma (ADCA) patients [6]. The refined region maps to chromosome 1p34 and includes MYCL1, TRIT1 (tRNA isopentenyltransferase 1), and MFSD2A (major facilitator superfamily domain containing 2). While the role of MYCL1 and TRIT1 in lung tumor growth and development has been studied [6,7], no information is available on MFSD2A. Thus, we addressed the functional role of MFSD2A in lung tumorigenesis.

## Results

### Downregulation of MFSD2A in lung cancer

**Figure 1:**
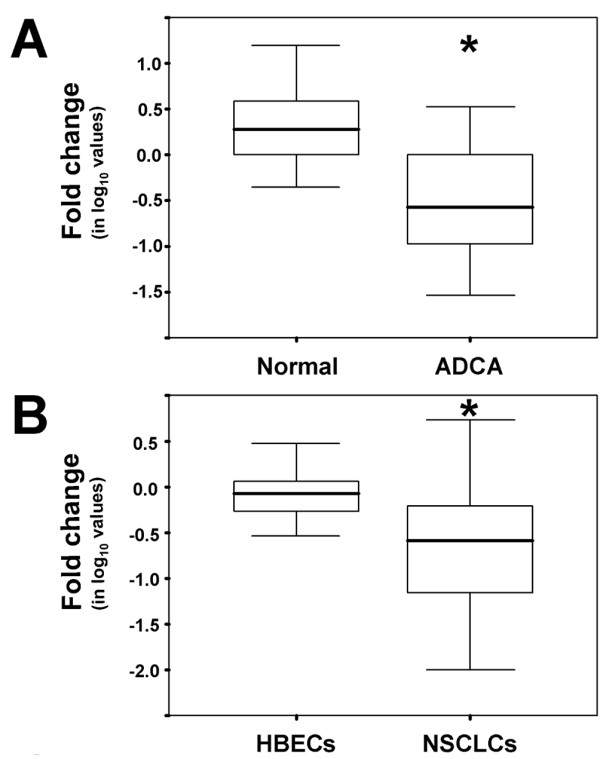
**MFSD2A expression is downregulated in human lung primary tumors and lung cancer cell lines**. (A) MFSD2A expression in 18 paired normal and tumor specimens obtained from lung adenocarcinoma patients. (B) MFSD2A expression in 20 human bronchial epithelial cell (HBEC) and 47 non small cell lung cancer (NSCLC) cell lines. Box boundaries indicate the 25th and 75th percentile, line within each box indicates the median, and error bars indicate the 10^th ^and 90^th ^percentile.

Based on our previous finding of MFSD2A downregulation in a pool of lung tumor specimens [7], we extended the analysis to 18 individual samples of lung ADCA tumors and corresponding benign adjacent tissue. MFSD2A mRNA levels were strongly downregulated (2- to 80-fold) in 17/18 tumors with an overall 5-fold decrease in ADCA as compared to normal lung specimens (*P *= 5.1e-05) (Fig. 1A). Statistical analysis showed no association with sex, age at diagnosis, or clinical stage (data not shown). It was not possible to evaluate association with smoking status since 17/18 patients were smokers.

Measurement of MFSD2A mRNA levels in NSCLC cell lines and normal human bronchial epithelial cell (HBEC) lines (Additional file 1), normalizing the data to the average expression of HBECs, revealed downregulation of MFSD2A (2- to 44-fold) in 33/47 (70%) NSCLC cell lines but only in 4/20 (20%) HBEC lines (Fig. 1B).

**Figure 2:**
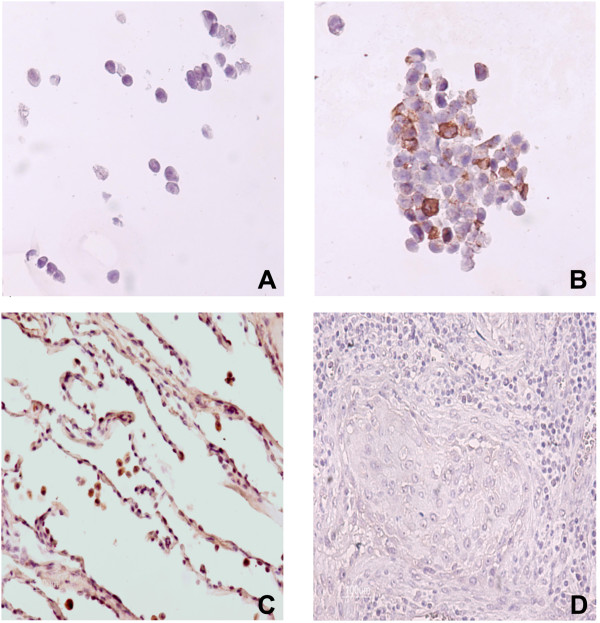
**Immunohistochemical analysis of MFSD2A protein**. No MFSD2A protein was detected in empty vector-transfected HEK-293T cells (negative control) (panel A), whereas a clear and mainly cytoplasmic staining pattern was observed in MFSD2A-transfected cells (panel B). In normal lung tissue, immunoreactivity is detected in lung alveolar cells (panel C), whereas almost no detectable staining is observed in lung ADCA (panel D).

To identify the lung cell types expressing MFSD2A protein, we have assayed by immunohistochemistry specimens of normal lung tissue and lung ADCA. Using non-transfected cells as a negative control (Fig. 2A), we confirmed the specificity of MFSD2A antibody on transfected cells over-expressing MFSD2A (Fig. 2B). In normal lung tissue, immunostaining of MFSD2A was observed in epithelial cells (Fig. 2C), whereas lung ADCAs showed no detectable MFSD2A protein levels (Fig. 2D) in agreement with the data of mRNA expression.

### A specific transcriptional profile is associated with MFSD2A over-expression

Changes in gene expression dependent on or correlated to MFSD2A were studied using two different approaches both based on microarray analysis.

Class comparison analysis of the expression profile of lung tumor cells transiently transfected with MFSD2A identified 460 genes whose expression differed significantly after MFSD2A exogenous expression (*P *< 0.001, FDR < 0.03) (Additional file 2). Microarray results were validated on a subset of 15 genes by real-time PCR (rho = 0.83, P < 0.0001). Analysis with the DAVID (Database for Annotation, Visualization and Integrated Discovery) Functional Annotation Tool [8] pointed to four main gene ontology categories: regulation of transcription, mitosis, apoptosis, and cell cycle.

Comparison of microarray data for NSCLC cell lines showing the highest and lowest MFSD2A mRNA levels identified 200 genes displaying at least a 4-fold difference (P < 0.005; Additional file 3). Most of the MFSD2A-correlated genes control developmental processes, neurodevelopment, cell motility, and adhesion.

### MFSD2A controls tumor growth and G0/G1 phase of lung cells

**Figure 3:**
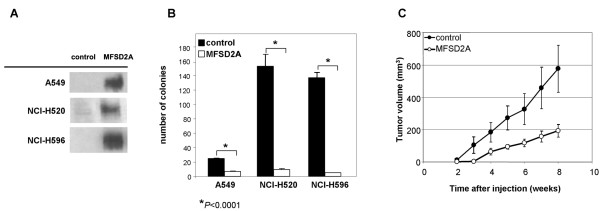
**Exogenous expression of MSFD2 inhibits growth of NSCLC cell lines *in vitro *and *in vivo***. (A) Western blots showing MFSD2A expression in MFSD2A-transfected and empty vector-transfected cell lines. (B) Colony number in each cell lines tested by colony formation assay. Data are given as mean ± SE of 6 independent replicas. (C) *In vivo *growth of MFSD2A-transfected and control (empty vector-transfected) A549 cells. Data are given as mean ( ± SE) tumor volume in 5 mice (control) or from two groups of 5 mice each injected with two independent MFSD2A clones.

NSCLC cell lines A549 (lung carcinoma), NCI-H520 (squamous cell carcinoma), and NCI-H596 (adenosquamous carcinoma), expressing MFSD2A at very low levels, were stably transfected to over-express MFSD2A (Fig. 3A) and showed a 4-, 16-, and 27-fold reduction in colony number in a colony formation assay, respectively, as compared to their empty vector-transfected counterparts (*P *< 0.0001) (Fig. 3B).

Analysis of tumor growth in nude mice injected subcutaneously with control or MFSD2A-transfected A549 cells revealed xenograft tumors at 2 weeks after injection in all control cell-treated mice, whereas tumors were measurable in all mice treated with MFSD2A-transfected cells only at 5 weeks post-injection. At the end of the observation period, tumors grown from MFSD2A-expressing cells were, on average, ~3-fold smaller than those from control cells (*P *< 0.0001) (Fig. 3C).

**Figure 4:**
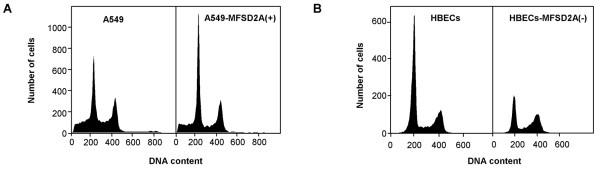
**MFSD2A affects G1 phase of lung tumor and normal cells**. Cell cycle profile of A549 cells over-expressing MFSD2A (A) and of normal HBECs after MFSD2A silencing (B).

FACS analysis of A549 cells stably expressing MFSD2A showed a significant increase in the G1 phase fraction and a reduction in DNA synthetic activity in S phase (Fig. 4, left panel). Analysis of three replica experiments showed that the proportion of cells accumulating at the G1 peak increased from 33% to 47% in MFSD2A-over-expressing cells, whereas the proportion of cells in S phase decreased from 18% to 13%.

To confirm this finding we studied changes in the cell cycle profile of normal human bronchial epithelial cells (HBECs) transfected with siRNAs targeting MFSD2A. Quantitative real-time PCR showed that 3 of 5 oligos (5'-catggagagtaacctcatcat-3', 5'-gagtgtcactgggcatttcta-3', 5'-ccactgtgaatatgccaagga-3') induced >3-fold reduction in MFSD2A mRNA levels. Low MFSD2A expression resulted in fewer cells accumulated in G1 as compared to control cells (from 59% to 41%) (Fig. 4, right panel), whereas distribution of cells in S and G2/M phases varied among the different samples (three independent replicates). Overall statistical analysis showed a significant effect of MFSD2A category of expression (over-expression or silencing) on modulation of cells accumulating in G1 fraction (P < 0.0001), with significant differences between the two cell lines (P = 0.003), and no significant interaction between MFSD2A expression and cell lines.

### MFSD2A over-expression modulates cell adhesion and cell migration

**Figure 5:**
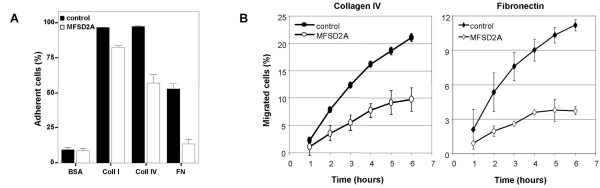
**A549 cells stably expressing MFSD2A have reduced adhesive and migratory properties**. (A) Adhesion of MFSD2A-expressing and control A549 cells on membranes coated with different substrates. Data are given as mean ± SE in 2 independent experiments, with 8 replicas in each experiment. (B) Migration of control and MFSD2A-expressing cells. Data are given as mean ± SE in 2 independent experiments, with 8 replicas in each experiment.

Adhesion and migration of stably transfected A549 cells were tested on membranes coated with different extracellular matrix (ECM) substrates (collagen I, collagen IV, and fibronectin) as compared to BSA-coated negative control membranes. Adhesion of MFSD2A-over-expressing cells was reduced by ~15% on collagen I, by ~40% on collagen IV, and by ~70% on fibronectin-coated membranes (Fig. 5A). Statistical analysis indicated a significant gene and substrate effect as well as a significant gene-substrate interaction (*P *< 0.0001).

The effects of MFSD2A expression on cancer cell migration were evaluated in a time-course experiment with the same ECM substrates used in the adhesion assay. No difference was seen in migration on collagen I-coated membranes, which also displayed the smallest effects in adhesion experiments (not shown). In contrast, the different migratory behavior of MFSD2A-transfected cells on collagen IV- and fibronectin-coated membranes was already apparent at early time points and was maintained throughout the whole experiment (Fig. 5B). At the final time point of the experiment, migration was reduced by ~60% on collagen IV and by ~80% on fibronectin. Statistical analysis indicated a significant difference between control and MFSD2A cells (*P *< 0.0001) as well as between substrates (collagen-IV and fibronectin) (*P *< 0.001).

To characterize the genes responsible for modulation of cell adhesion and cell migration, we have measured the transcript levels of genes involved in extracellular matrix structure and remodeling. Analysis of two independent A549 cell clones stably transfected with MFSD2A showed that 41 of 66 informative gene targets were downregulated (P < 0.05) as compared to A549 control cells and 25 of these genes showed a >3-fold change (Table 1).

**Table 1:** List of extracellular matrix genes >3-fold downregulated by MFSD2A over-expression in stable-transfected A549 cells.

Gene symbol	Gene name	Fold-change *	*P*
ADAMTS1	ADAM metallopeptidase with thrombospondin type 1 motif, 1	0.25	1.13E-07
CDH1	cadherin 1, type 1, E-cadherin (epithelial)	0.07	2.57E-02
COL11A1	collagen, type XI, alpha 1	0.10	2.60E-02
COL12A1	collagen, type XII, alpha 1	0.09	1.01E-03
COL4A2	collagen, type IV, alpha 2	0.25	1.78E-07
COL5A1	collagen, type V, alpha 1	0.25	1.22E-07
COL6A1	collagen, type VI, alpha 1	0.18	4.30E-03
COL7A1	collagen, type VII, alpha 1	0.25	3.60E-07
CTGF	connective tissue growth factor	0.07	1.56E-08
CTNND1	catenin (cadherin-associated protein), delta 1	0.32	1.05E-02
FN1	fibronectin 1	0.16	2.53E-02
ICAM1	intercellular adhesion molecule 1	0.25	2.18E-07
ITGA2	integrin, alpha 2 (CD49B, alpha 2 subunit of VLA-2 receptor)	0.18	3.52E-03
ITGA7	integrin, alpha 7	0.19	3.96E-03
LAMB3	laminin, beta 3	0.15	2.46E-02
MMP1	matrix metallopeptidase 1 (interstitial collagenase)	0.33	7.35E-03
MMP10	matrix metallopeptidase 10 (stromelysin 2)	0.27	1.84E-02
MMP14	matrix metallopeptidase 14 (membrane-inserted)	0.16	2.17E-03
MMP15	matrix metallopeptidase 15 (membrane-inserted)	0.25	3.21E-02
SELL	selectin L	0.19	4.59E-03
SPARC	secreted protein, acidic, cysteine-rich (osteonectin)	0.08	1.08E-02
TGFBI	transforming growth factor, beta-induced, 68 kDa	0.25	3.22E-08
THBS1	thrombospondin 1	0.19	3.82E-03
THBS3	thrombospondin 3	0.31	9.78E-03
TIMP3	TIMP metallopeptidase inhibitor 3	0.02	6.21E-05

* With respect to untransfected A549 cells.

## Discussion

In this study, most of the primary lung tumors and NSCLC cell lines examined showed significantly lower MFSD2A expression as compared to their normal counterparts. The mechanisms underlying the inhibition of MFSD2A expression are unclear, although our preliminary experiments indicating restoration of MFSD2A expression in NSCLC cell lines upon treatment with 5'-azacytidine, a methyltransferase inhibitor and demethylating agent, suggest a role for methylation. Other possible mechanisms of gene inactivation, such as the presence of somatic mutations or the influence of microRNAs, remain to be investigated.

Over-expression of MFSD2A in transfected lung cancer cell lines was associated with reduced clonogenicity *in vitro *and diminished tumorigenicity *in vivo*, an effect due likely to the ability of MFSD2A to block the cell cycle in the G1 phase and to impair adhesive and migratory properties. Indeed, microarray data indicated that MFSD2A regulates expression of genes controlling cell cycle progression and correlates with expression of genes affecting adhesion and motility. Although microarray analysis also pointed to regulation of apoptotic genes, the FACS profile of MFSD2A-transfected cells revealed no increase in the sub-G1 phase fraction, no change was observed after annexin V staining, and neither PARP nor caspase cleavage was detected (data not shown).

MFSD2A has recently been described as the human receptor for syncytin-2, a retrovirus-derived protein mediating fusion of placental trophoblasts; however, *in silico *analysis predicts that the primary function of the gene is in transport of carbohydrates [9]. The presence of this gene within the MYCL1 linkage disequilibrium block associated with differences in the survival of lung cancer patients [6] suggests a role for MFSD2A in controlling predisposition to lung cancer progression, although sequencing of coding regions identified no functional polymorphism in MFSD2A that could account for this effect [6]. Studies addressing the possible existence of genetic variations in the promoter region and their role in populations of different ethnicities will shed light on the potential role of MFSD2A in modifying lung cancer progression.

## Conclusion

Based on the present findings we can conclude that MFSD2A is a novel suppressor gene in lung cancer acting on tumor growth and development through control of cell cycle profile, matrix attachment, and cell motility.

## Methods

### Lung Primary Samples and Cell lines

Matched specimens of normal lung parenchyma and lung adenocarcinoma tissue were obtained from patients who underwent lobectomy at Istituto Nazionale Tumori (Milan, Italy). Normal human bronchial epithelial cells (HBECs) and lung tumor cell lines were available at the tissue culture repository of the Hamon Center for Therapeutic Oncology Research, UTSW Medical Center (Dallas, TX) [10]. Cell lines that have been used are listed in Additional file 1.

### Quantitative real-time PCR

Total RNA was extracted with the RNeasy Midi kit (Qiagen, Valencia, CA) and reverse-transcribed with either the SuperScript First-Strand Synthesis System (Invitrogen, Carlsbad, CA). MFSD2A mRNA expression was analyzed using TaqMan gene expression assays (Applied Biosystems, Foster City, CA): MFSD2A (Hs00293017_m1), HPRT1 (Hs99999909_m1), and GAPDH (4352934E).

Intron-spanning primers were designed to validate microarray results of NCI-H520 transfected cells for the following genes: DDIT3, DNAJB9, ELMO3, HRK, IRF1, LAMP3, MX1, NUPR1, PARL, PPP1R15A, RYR1, S100P, TCTA, UHMK1, WIPI1, and HPRT1 (housekeeping control). Amplification mixtures contained cDNA template, Power SYBR^® ^Green PCR Master Mix (Applied Biosystems), and gene-specific PCR primers (sequences of oligonucleotide primers are available upon request).

For the expression levels analysis of genes involved in cell adhesion and cell migration pathways, we have used the TaqMan Array Human Extracellular Matrix & Adhesion Molecules 96-well plate (Applied Biosystems), according to the manufacturer's instructions.

Relative expression values were calculated using the comparative Ct method.

### Microarray analysis

Gene expression profile of NCI-H520 cells transfected with recombinant MFSD2A or empty control vector (4 replicas/each) was analyzed using the Human-8 v3 Expression BeadChips (Illumina Inc., San Diego, CA, USA). Intensity values of each hybridization were quality-checked and the data set was normalized using a cubic spline algorithm, with BeadStudio Version 3 software. A P-value < 0.05 was set as a cutoff to filter reliably detected genes.

Gene expression profile of the 47 NSCLC cell lines was analyzed on the Affymetrix GeneChips HG-U133A and HG-U133B (together 44,928 elements; 23,583 unique genes) according to the manufacturer's protocol. Intensity values were quality-checked and the data set was quantile-normalized.

### MFSD2A over-expression and silencing

MFSD2A full-length transcript (NM_032793) was amplified from a pool of human lung cDNAs (primers 5'-ggtcatggccaaaggagaa-3' and 5'-gaggatgctagccagctctgtg-3'), cloned in pEF6/V5-His TOPO vector (Invitrogen), and sequenced. Cell lines were transiently transfected using FuGENE HD Transfection Reagent (Roche, Basel, Switzerland). Stable transfectant clones were obtained after selection with 5 (A549) or 2 (NCI-H596, NCI-H520) μg/ml of blasticidin (Invitrogen) for 2 weeks.

siRNAs targeted against MFSD2A (5'-gcttcacaaagtgccaaccat-3', 5'-catggagagtaacctcatcat-3', 5'-gagtgtcactgggcatttcta-3', 5'-cacggcccatacatcaaactt-3', 5'-ccactgtgaatatgccaagga-3') were purchased from Qiagen. HBECs cells, which express endogenous MFSD2A, were transfected with 25 nM siRNA oligonucleotides using Oligofectamine (Invitrogen) and harvested after 96 hours for quantitative real-time PCR or cell cycle analysis.

### Western blots and immunohistochemistry

MFSD2A expression in stably transfected cells was detected by Western blotting with anti-V5-HRP antibody (Invitrogen).

Samples of paraffin-embedded sections of MFSD2A- or empty vector-transfected HEK-293T cells, lung ADCA tissue, and surrounding normal lung tissue were used to prepare histological sections that were immunostained using standard methods after antigen retrieval performed in 0.07 M citrate buffer (pH = 6) at 95°C for 10 min. Mouse polyclonal anti-MFSD2A antibody (H00084879-B01P; Abnova, Taipei City, Taiwan) was used at a 1:170 dilution. Immunoreactive signals were detected with ChemMate DAB (Dako, Glostrup, Denmark).

### In vitro assays

Colony formation assay was carried out in stably transfected A549, NCI-H520, and NCI-H596 cells (6 replicas). After 2 weeks of selection, colonies were methanol-fixed, stained with 10% Giemsa, and manually counted.

Cell adhesion was measured using CAFCA (Centrifugal Assay for Fluorescence based Cell Adhesion) assay [11]. Briefly, six well strips were coated with different substrates from Becton-Dickinson (Falcon, Milan, Italy). Cells were labeled with the vital fluorochrome calcein AM (Invitrogen) for 15 minutes at 37°C and aliquoted into the bottom CAFCA miniplates, which were centrifuged to synchronize the contact of the cells with the substrate. The miniplates were incubated for 20 minutes at 37°C and mounted together with a similar CAFCA miniplate to create communicating chambers for reverse centrifugation. The relative number of cells bound to the substrate (i.e. remaining in the wells of the bottom miniplates) and cells that failed to bind to the substrate (i.e. remaining in the wells of the top miniplates) was estimated by top/bottom fluorescence detection with GENios Plus microplate fluorometer (TECAN, Italy). Percentage of adherent cells was determined 8 hours after plating.

Cell migration in response to extracellular matrix substrates was assessed by FATIMA (Fluorescence-Assisted Transmigration Invasion and Motility) assay as described [11]. Briefly, membranes of HTS FluoroBlokTM transwell inserts with 8 μm pores (Becton-Dickinson, Falcon) were coated on the underside with various ECM molecules at 4°C and blocked with 1% BSA. Cells were fluorescently tagged with 5 μg/ml DiI lipophilic dye (Invitrogen) and plated in the upper chamber. Migration was monitored at every hour by independent fluorescence detection from the top (corresponding to non-transmigrated cells) and bottom (corresponding to transmigrated cells) side of the membrane with GENios Plus microplate fluorometer (TECAN).

Cell cycle analysis of stably transfected cells was performed on a FACScan (BD Biosciences, San Jose, CA) or FACSCalibur flow cytometer (BD Biosciences). MFSD2A- or empty vector-transfected cells were harvested and fixed in 70% EtOH over night at 4°C. Cells were then incubated in 500 μl of buffered propidium iodide (PI) staining solution containing 0.05% Triton X-100 (Sigma-Aldrich), 0.1 mg/ml RNase A (Millipore), and 50 g/ml PI (Sigma-Aldrich) in PBS for 30 min at 37°C. Cells were briefly spun down and resuspended in PBS. Flow cytometry data were analyzed using FlowJo software (TreeStar, Ashland, OR) with the aid of the Watson modeling algorithm.

### In vivo tumor growth assay

Adult female CD-1 nude mice (purchased from Charles River, Calco, Italy) were injected subcutaneously with 4 × 10^6 ^A549 control or MFSD2A-transfected cells (2 independent clones). Tumor size was measured weekly by calipers and animals were sacrificed 8 weeks after injection.

### Statistical analysis

Genes differentially expressed in the two classes of either vector- or MFSD2A-transfected cells were identified using random variance t-statistics [12]. Microarray data were analyzed using BRB ArrayTools developed by Dr. Richard Simon and Amy Peng Lam http://linus.nci.nih.gov/BRB-ArrayTools.html (Illumina experiment) or using in-house Visual Basic software MATRIX 1.4 and the Bioconductor R package affy (Affymetrix experiment). Functional annotation in gene ontology (GO) categories was carried out with the DAVID Functional Annotation Tools [8]. Differences in quantitative measures were assessed by analysis of variance. Correlations between expression levels were expressed by the Spearman's correlation coefficient. Cell cycle parameters by MFSD2A categories of expression (high, low) were analyzed using the generalized linear model, family = quasibinomial, procedure.

## Competing interests

The authors declare that they have no competing interests.

## Authors' contributions

All authors read and approved the final manuscript.

MS carried out the molecular genetic studies and drafted the manuscript, FSF contributes with the design of the study and with the molecular genetics studies, FC contributed with *in vitro *functional assays, JPS contributed with cell cycle profile analyses, DSS contributed with *in vitro *functional assays, LG contributed with statistical analyses of data from normal and cancer cell lines, PS contributed with *in vitro *functional assays, JDM supervised the study and contributed to the manuscript preparation, TAD designed and coordinated the study and drafted the manuscript.

## Supplementary Material

Additional file 1 List of human lung cell lines that have been used. Click here for file

Additional file 2 **List of genes differentially expressed between MFSD2A- and vector-transfected NCI-H520 cells**. The gene expression profile of NSCLC line NCI-H520 transiently transfected cells (4 replicas) was analyzed using the Human-8 v3 Expression BeadChips (Illumina Inc., San Diego, CA). The data set was normalized using a cubic spline algorithm, with BeadStudio Version 3 software. A P-value < 0.05 was set as a cutoff to filter reliably detected genes. Click here for file

Additional file 3 **List of genes differentially expressed between lung cancer cell lines by MFSD2A mRNA levels**. The gene expression profile of 47 NSCLC cell lines was analyzed on the Affymetrix GeneChips HG-U133A and HG-U133B. Arrays from both types were pooled and normalized to their 100 common control genes. Groups of samples were compared by calculating log_2 _ratios for each gene. A T-test P-value < 0.005 was set as a cutoff to select differentially expressed genes. Click here for file
