# Supplementary material for: MFSD2A is a novel lung tumor suppressor gene modulating cell cycle and matrix attachment
Source: Mol Cancer. 2010 Mar 17;9:62. doi: 10.1186/1476-4598-9-62 (PMC2846890; doi:10.1186/1476-4598-9-62)
Supplement: Additional file 1 — List of human lung cell lines that have been used. [file 1476-4598-9-62-S1.DOC]

| Additional file 1. List of human lung cell lines that have been used. | |
| --- | --- |
| Cell line | Histotype |
| HBEC11KT | Human bronchial epithelial cell |
| HBEC12KT | Human bronchial epithelial cell |
| HBEC13KT | Human bronchial epithelial cell |
| HBEC14KT | Human bronchial epithelial cell |
| HBEC15KT | Human bronchial epithelial cell |
| HBEC16KT | Human bronchial epithelial cell |
| HBEC24KT | Human bronchial epithelial cell |
| HBEC27KT | Human bronchial epithelial cell |
| HBEC28KT | Human bronchial epithelial cell |
| HBEC2KT | Human bronchial epithelial cell |
| HBEC30KT | Human bronchial epithelial cell |
| HBEC34KT | Human bronchial epithelial cell |
| HBEC35KT | Human bronchial epithelial cell |
| HBEC36KT | Human bronchial epithelial cell |
| HBEC37KT | Human bronchial epithelial cell |
| HBEC3KT | Human bronchial epithelial cell |
| HBEC4KT | Human bronchial epithelial cell |
| HBEC5KT | Human bronchial epithelial cell |
| HBEC6KT | Human bronchial epithelial cell |
| HBEC7KT | Human bronchial epithelial cell |
| A549 | Adenocarcinoma |
| H1355 | Adenocarcinoma |
| H1395 | Adenocarcinoma |
| H1437 | Adenocarcinoma |
| H1648 | Adenocarcinoma |
| H1650 | Adenocarcinoma |
| H1666 | Adenocarcinoma |
| H1792 | Adenocarcinoma |
| H1819 | Adenocarcinoma |
| H1975 | Adenocarcinoma |
| H1993 | Adenocarcinoma |
| H2009 | Adenocarcinoma |
| H2087 | Adenocarcinoma |
| H23 | Adenocarcinoma |
| H2347 | Adenocarcinoma |
| H322 | Adenocarcinoma |
| H3255 | Adenocarcinoma |
| H358 | Adenocarcinoma |
| H441 | Adenocarcinoma |
| H820 | Adenocarcinoma |
| HCC1195 | Adenocarcinoma |
| HCC1833 | Adenocarcinoma |
| HCC193 | Adenocarcinoma |
| HCC2279 | Adenocarcinoma |
| HCC4006 | Adenocarcinoma |
| HCC461 | Adenocarcinoma |
| HCC515 | Adenocarcinoma |
| HCC78 | Adenocarcinoma |
| HCC827 | Adenocarcinoma |
| H596 | Adenosquamous cell lung carcinoma |
| H1155f | Large cell lung carcinoma |
| H1299 | Large cell lung carcinoma |
| H2126 | Large cell lung carcinoma |
| H460 | Large cell lung carcinoma |
| H661 | Large cell lung carcinoma |
| HCC1359 | Large cell lung carcinoma |
| Calu-1 | Non-small cell lung carcinoma |
| Calu-6 | Non-small cell lung carcinoma |
| H2882 | Non-small cell lung carcinoma |
| H2887 | Non-small cell lung carcinoma |
| H3122 | Non-small cell lung carcinoma |
| HCC1171 | Non-small cell lung carcinoma |
| HCC2935 | Non-small cell lung carcinoma |
| HCC366 | Non-small cell lung carcinoma |
| HCC44 | Non-small cell lung carcinoma |
| H157 | Squamous cell lung carcinoma |
| H520 | Squamous cell lung carcinoma |
| HCC15 | Squamous cell lung carcinoma |
| HCC95 | Squamous cell lung carcinoma |
