# Supplementary material for: MFSD2A is a novel lung tumor suppressor gene modulating cell cycle and matrix attachment
Source: Mol Cancer. 2010 Mar 17;9:62. doi: 10.1186/1476-4598-9-62 (PMC2846890; doi:10.1186/1476-4598-9-62)
Supplement: Additional file 2 — List of genes differentially expressed between MFSD2A- and vector-transfected NCI-H520 cells. The gene expression profile of NSCLC line NCI-H520 transiently transfected cells (4 replicas) was analyzed using the Human-8 v3 Expression BeadChips (Illumina Inc., San Diego, CA). The data set was normalized using a cubic spline algorithm, with BeadStudio Version 3 software. A P-value < 0.05 was set as a cutoff to filter reliably detected genes. [file 1476-4598-9-62-S2.DOC]

| Additional file 2. List of genes differentially expressed between MFSD2A- and vector-transfected NCI-H520 cells (P<0.001, FDR<0.03). | | | |
| --- | --- | --- | --- |
| Gene symbol | Gene name | Gene accession # | MFDS2/vector ratio |
| WIPI1 | WD repeat domain, phosphoinositide interacting 1 | NM_017983 | 3.2 |
| NRIP1 | nuclear receptor interacting protein 1 | NM_003489 | 3.0 |
| INHBE | inhibin, beta E | NM_031479 | 2.9 |
| RETNLB | resistin like beta | NM_032579 | 2.9 |
| S100P | S100 calcium binding protein P | NM_005980 | 2.6 |
| IRF7 | interferon regulatory factor 7 | NM_004029 | 2.5 |
| GDF15 | growth differentiation factor 15 | NM_004864 | 2.5 |
| ULBP1 | UL16 binding protein 1 | NM_025218 | 2.5 |
| TTC25 | tetratricopeptide repeat domain 25 | NM_031421 | 2.5 |
| CKMT1B | creatine kinase, mitochondrial 1B, nuclear gene encoding mitochondrial protein | NM_020990 | 2.4 |
| NUPR1 | nuclear protein 1 | NM_001042483 | 2.4 |
| ISGF3G | interferon-stimulated transcription factor 3, gamma 48kDa | NM_006084 | 2.3 |
| IRXL1 | mohawk homeobox | NM_173576 | 2.2 |
| ASS | argininosuccinate synthetase | NM_000050 | 2.2 |
| TRIB3 | tribbles homolog 3 | NM_021158 | 2.2 |
| G1P3 | interferon, alpha-inducible protein, | NM_002038 | 2.1 |
| MX1 | myxovirus resistance 1, interferon-inducible protein p78 | NM_002462 | 2.1 |
| ZNF555 | zinc finger protein 555 | NM_152791 | 2.1 |
| STMN4 | stathmin-like 4 | NM_030795 | 2.1 |
| LAMP3 | lysosomal-associated membrane protein 3 | NM_014398 | 2.0 |
| HIST1H2AC | histone cluster 1, H2ac | NM_003512 | 2.0 |
| HIST1H4H | histone cluster 1, H4h | NM_003543 | 2.0 |
| HYPE | Huntingtin interacting protein E | NM_007076 | 2.0 |
| ASS1 | argininosuccinate synthetase 1 | NM_054012 | 2.0 |
| STAG3 | stromal antigen 3 | NM_012447 | 2.0 |
| HIST2H4A | histone cluster 2, H4a | NM_003548 | 2.0 |
| C17orf28 | chromosome 17 open reading frame 28 | NM_030630 | 2.0 |
| HRK | harakiri, BCL2 interacting protein | NM_003806 | 2.0 |
| DNAJB9 | DnaJ homolog, subfamily B, member 9 | NM_012328 | 2.0 |
| HERPUD1 | homocysteine-inducible, endoplasmic reticulum stress-inducible, ubiquitin-like domain member 1 | NM_001010990 | 1.9 |
| DDIT4 | DNA-damage-inducible transcript 4 | NM_019058 | 1.8 |
| GADD45G | growth arrest and DNA-damage-inducible, gamma | NM_006705 | 1.8 |
| SLC1A4 | solute carrier family 1, member 4 | NM_003038 | 1.8 |
| DDIT3 | DNA-damage-inducible transcript 3 | NM_004083 | 1.8 |
| NIP7 | nuclear import 7 homolog | NM_016101 | 1.8 |
| ANG | angiogenin, ribonuclease, RNase A family, 5 | NM_001145 | 1.8 |
| P8 | p8 protein | NM_012385 | 1.8 |
| PPP1R15A | protein phosphatase 1, regulatory subunit 15A | NM_014330 | 1.8 |
| SLC6A9 | solute carrier family 6, member 9 | NM_201649 | 1.8 |
| ZNF222 | zinc finger protein 222 | NM_013360 | 1.7 |
| KLF2 | Kruppel-like factor 2 | NM_016270 | 1.7 |
| RNF148 | ring finger protein 148 | NM_198085 | 1.7 |
| FBXO32 | F-box protein 32 | NM_058229 | 1.7 |
| ADM2 | adrenomedullin 2 | NM_024866 | 1.7 |
| FAM83F | family with sequence similarity 83, member F | NM_138435 | 1.7 |
| EFNA1 | ephrin-A1 | NM_004428 | 1.7 |
| SIRT4 | sirtuin 4 | NM_012240 | 1.7 |
| RN7SK | RNA, 7SK, nuclear on chromosome 6 | NR_001445 | 1.7 |
| TNFAIP3 | tumor necrosis factor, alpha-induced protein 3 | NM_006290 | 1.7 |
| CHAC1 | ChaC, cation transport regulator homolog 1 | NM_024111 | 1.7 |
| NGFRAP1L1 | NGFRAP1-like 1 | NM_001012978 | 1.7 |
| ISL2 | ISL LIM homeobox 2 | NM_145805 | 1.7 |
| ATF3 | activating transcription factor 3 | NM_001040619 | 1.7 |
| ABHD14A | abhydrolase domain containing 14A | NM_015407 | 1.6 |
| IRF1 | interferon regulatory factor 1 | NM_002198 | 1.6 |
| ZNF467 | zinc finger protein 467 | NM_207336 | 1.6 |
| CBLB | Cas-Br-M ecotropic retroviral transforming sequence b | NM_170662 | 1.6 |
| ISG20 | interferon stimulated exonuclease gene 20kDa | NM_002201 | 1.6 |
| RELB | v-rel reticuloendotheliosis viral oncogene homolog B, nuclear factor of kappa light polypeptide gene enhancer in B-cells 3 | NM_006509 | 1.6 |
| LOC389286 | similar to FKSG62 | NM_001018022 | 1.6 |
| CARS | cysteinyl-tRNA synthetase | NM_001751 | 1.6 |
| RYR1 | ryanodine receptor 1 | NM_000540 | 1.6 |
| ZSWIM3 | zinc finger, SWIM-type containing 3 | NM_080752 | 1.6 |
| SELM | selenoprotein M | NM_080430 | 1.6 |
| PLEKHF1 | pleckstrin homology domain containing, family F member 1 | NM_024310 | 1.6 |
| MEF2A | myocyte enhancer factor 2A | NM_005587 | 1.6 |
| CALCB | calcitonin-related polypeptide, beta | NM_000728 | 1.6 |
| MGC14376 | hypothetical protein MGC14376 | NM_001001870 | 1.6 |
| CRELD1 | cysteine-rich with EGF-like domains 1 | NM_015513 | 1.6 |
| NTF3 | neurotrophin 3 | NM_002527 | 1.6 |
| PNPLA8 | patatin-like phospholipase domain containing 8 | NM_015723 | 1.6 |
| KLF5 | Kruppel-like factor 5 | NM_001730 | 1.6 |
| PCK2 | phosphoenolpyruvate carboxykinase 2, nuclear gene encoding mitochondrial protein | NM_001018073 | 1.6 |
| PPL | periplakin | NM_002705 | 1.5 |
| CBX8 | chromobox homolog 8 | NM_020649 | 1.5 |
| GOLGB1 | golgi autoantigen, golgin subfamily b, macrogolgin, 1 | NM_004487 | 1.5 |
| MGC15875 | hypothetical protein LOC85007, isoform 2 | NM_032921 | 1.5 |
| CKMT1A | creatine kinase, mitochondrial 1A, nuclear gene encoding mitochondrial protein | NM_001015001 | 1.5 |
| WARS | tryptophanyl-tRNA synthetase | NM_213646 | 1.5 |
| MGC99813 | similar to RIKEN cDNA A230078I05 gene | NM_001005209 | 1.5 |
| AXUD1 | AXIN1 up-regulated 1 | NM_033027 | 1.5 |
| TES | testis derived transcript | NM_152829 | 1.5 |
| C9orf85 | chromosome 9 open reading frame 85 | NM_182505 | 1.5 |
| IFITM2 | interferon induced transmembrane protein 2 | NM_006435 | 1.5 |
| WDR19 | WD repeat domain 19 | NM_025132 | 1.5 |
| NBLA10383 | putative protein product of Nbla10383 | NM_173622 | 1.5 |
| SH2B1 | SH2B adaptor protein 1 | NM_015503 | 1.5 |
| ZNF225 | zinc finger protein 225 | NM_013362 | 1.5 |
| ZNF289 | zinc finger protein 289, ID1 regulated | NM_032389 | 1.5 |
| PLGLB1 | plasminogen-like B1 | NM_001032392 | 1.5 |
| SEDLP | spondyloepiphyseal dysplasia, late, pseudogene on chromosome 19 | NR_002166 | 1.5 |
| ZFAND2A | zinc finger, AN1-type domain 2A | NM_182491 | 1.5 |
| HIST1H1C | histone cluster 1, H1c | NM_005319 | 1.5 |
| RAGE | renal tumor antigen | NM_014226 | 1.5 |
| IL23A | interleukin 23, alpha subunit p19 | NM_016584 | 1.5 |
| GFPT1 | glutamine-fructose-6-phosphate transaminase 1 | NM_002056 | 1.5 |
| HADH2 | hydroxyacyl-Coenzyme A dehydrogenase, type II | NM_004493 | 1.5 |
| ZNF433 | zinc finger protein 433 | NM_152602 | 1.5 |
| C21orf7 | chromosome 21 open reading frame 7 | NM_020152 | 1.5 |
| KCNG1 | potassium voltage-gated channel, subfamily G, member 1 | NM_002237 | 1.5 |
| DERL2 | Der1-like domain family, member 2 | NM_016041 | 1.5 |
| CXorf23 | chromosome X open reading frame 23 | NM_198279 | 1.5 |
| TMEM125 | transmembrane protein 125 | NM_144626 | 1.5 |
| RNF113A | ring finger protein 113A | NM_006978 | 1.5 |
| F25965 | lin-37 homolog | NM_019104 | 1.5 |
| PRIC285 | peroxisomal proliferator-activated receptor A interacting complex 285 | NM_033405 | 1.5 |
| GALR2 | galanin receptor 2 | NM_003857 | 1.4 |
| LARP6 | La ribonucleoprotein domain family, member 6 | NM_018357 | 1.4 |
| ARHGEF2 | rho/rac guanine nucleotide exchange factor 2 | NM_004723 | 1.4 |
| JMY | junction-mediating and regulatory protein | NM_152405 | 1.4 |
| PECR | peroxisomal trans-2-enoyl-CoA reductase | NM_018441 | 1.4 |
| CBX4 | chromobox homolog 4 | NM_003655 | 1.4 |
| KLF6 | Kruppel-like factor 6 | NM_001008490 | 1.4 |
| GABARAPL1 | GABA(A) receptor-associated protein like 1 | NM_031412 | 1.4 |
| XBP1 | X-box binding protein 1 | NM_001079539 | 1.4 |
| ZYX | zyxin | NM_003461 | 1.4 |
| TRIM5 | tripartite motif-containing 5 | NM_033034 | 1.4 |
| EVI5L | ecotropic viral integration site 5-like | NM_145245 | 1.4 |
| FLII | flightless I homolog | NM_002018 | 1.4 |
| GPR30 | G protein-coupled estrogen receptor 1 | NM_001039966 | 1.4 |
| ATG3 | ATG3 autophagy related 3 homolog | NM_022488 | 1.4 |
| C20orf31 | ER degradation enhancer, mannosidase alpha-like 2 | NM_018217 | 1.4 |
| GADD45B | growth arrest and DNA-damage-inducible, beta | NM_015675 | 1.4 |
| RPL14 | ribosomal protein L14 | NM_003973 | 1.4 |
| GPAA1 | GPAA1P anchor attachment protein 1 homolog | NM_003801 | 1.4 |
| ZNF34 | zinc finger protein 34 | NM_030580 | 1.4 |
| SEC24D | SEC24 related gene family, member D | NM_014822 | 1.4 |
| CEBPB | CCAAT/enhancer binding protein, beta | NM_005194 | 1.4 |
| CRELD2 | cysteine-rich with EGF-like domains 2 | NM_024324 | 1.4 |
| PLEKHH3 | pleckstrin homology domain containing, family H member 3 | NM_024927 | 1.4 |
| IFI35 | interferon-induced protein 35 | NM_005533 | 1.4 |
| FBXL20 | F-box and leucine-rich repeat protein 20 | NM_032875 | 1.4 |
| ZHX2 | zinc fingers and homeoboxes 2 | NM_014943 | 1.4 |
| PSPH | phosphoserine phosphatase | NM_004577 | 1.4 |
| CICE | cell death-inducing DFFA-like effector c pseudogene on chromosome 3 | NR_002786 | 1.4 |
| RWDD2 | RWD domain containing 2A | NM_033411 | 1.4 |
| STX5A | syntaxin 5A | NM_003164 | 1.4 |
| HIST2H2BF | histone 2, H2bf | NM_001024599 | 1.4 |
| TICAM2 | toll-like receptor adaptor molecule 2 | NM_021649 | 1.4 |
| TIPARP | TCDD-inducible poly(ADP-ribose) polymerase | NM_015508 | 1.4 |
| PMAIP1 | phorbol-12-myristate-13-acetate-induced protein 1 | NM_021127 | 1.4 |
| ASNS | asparagine synthetase | NM_133436 | 1.4 |
| PPIB | peptidylprolyl isomerase B | NM_000942 | 1.4 |
| CSNK1A1 | casein kinase 1, alpha 1 | NM_001892 | 1.4 |
| VCX-C | variably charged X-C | NM_001001888 | 1.4 |
| WDR45 | WD repeat domain 45 | NM_001029896 | 1.4 |
| ZNF16 | zinc finger protein 16 | NM_006958 | 1.4 |
| PLSCR1 | phospholipid scramblase 1 | NM_021105 | 1.4 |
| RPL32P3 | ribosomal protein L32 pseudogene 3 on chromosome 3 | NR_003111 | 1.4 |
| RFX3 | regulatory factor X, 3 | NM_134428 | 1.4 |
| NUCB2 | nucleobindin 2 | NM_005013 | 1.4 |
| KLF10 | Kruppel-like factor 10 | NM_005655 | 1.4 |
| DUSP16 | dual specificity phosphatase 16 | NM_030640 | 1.4 |
| DHRS9 | dehydrogenase/reductase member 9 | NM_005771 | 1.4 |
| SLC7A11 | solute carrier family 7, member 11 | NM_014331 | 1.4 |
| RRBP1 | ribosome binding protein 1 homolog 180kDa | NM_004587 | 1.4 |
| C12orf10 | chromosome 12 open reading frame 10 | NM_021640 | 1.4 |
| ZNF502 | zinc finger protein 502 | NM_033210 | 1.4 |
| TXNDC11 | thioredoxin domain containing 11 | NM_015914 | 1.4 |
| SCG2 | secretogranin II | NM_003469 | 1.4 |
| JARID1B | jumonji, AT rich interactive domain 1B | NM_006618 | 1.4 |
| SELS | selenoprotein S | NM_203472 | 1.4 |
| SPSB3 | splA/ryanodine receptor domain and SOCS box containing 3 | NM_080861 | 1.3 |
| YPEL5 | yippee-like 5 | NM_016061 | 1.3 |
| FOXJ2 | forkhead box J2 | NM_018416 | 1.3 |
| SIN3A | SIN3 homolog A, transcription regulator | NM_015477 | 1.3 |
| PGM3 | phosphoglucomutase 3 | NM_015599 | 1.3 |
| DCTN5 | dynactin 5 | NM_032486 | 1.3 |
| FLJ39575 | hypothetical protein FLJ39575 | NM_182597 | 1.3 |
| MGC10471 | coiled-coil domain containing 130 | NM_030818 | 1.3 |
| ARCN1 | archain 1 | NM_001655 | 1.3 |
| ZNF622 | zinc finger protein 622 | NM_033414 | 1.3 |
| WDSUB1 | WD repeat, sterile alpha motif and U-box domain containing 1 | NM_152528 | 1.3 |
| CLIPR-59 | CAP-GLY domain containing linker protein 3 | NM_015526 | 1.3 |
| RHOQ | ras homolog gene family, member Q | NM_012249 | 1.3 |
| BEXL1 | brain expressed X-linked-like 1 | NM_001080425 | 1.3 |
| COMMD1 | copper metabolism domain containing 1 | NM_152516 | 1.3 |
| CDK2AP2 | CDK2-associated protein 2 | NM_005851 | 1.3 |
| LOC285074 | hypothetical protein LOC285074 | NM_001012626 | 1.3 |
| LOC440348 | similar to nuclear pore complex interacting protein | NM_001018059 | 1.3 |
| TRIM11 | tripartite motif-containing 11 | NM_145214 | 1.3 |
| CD63 | CD63 antigen | NM_001780 | 1.3 |
| U2AF1L2 | U2(RNU2) small nuclear RNA auxiliary factor 1-like 2 | NM_005089 | 1.3 |
| CIR | CBF1 interacting corepressor | NM_004882 | 1.3 |
| MRPL20 | mitochondrial ribosomal protein L20, nuclear gene encoding mitochondrial protein | NM_017971 | 1.3 |
| AHR | aryl hydrocarbon receptor | NM_001621 | 1.3 |
| ENTPD4 | ectonucleoside triphosphate diphosphohydrolase 4 | NM_004901 | 1.3 |
| C14orf122 | chromosome 14 open reading frame 122 | NM_016049 | 1.3 |
| TMCO3 | transmembrane and coiled-coil domains 3 | NM_017905 | 1.3 |
| FKBP11 | FK506 binding protein 11, 19 kDa | NM_016594 | 1.3 |
| SELK | selenoprotein K | NM_021237 | 1.3 |
| LPXN | leupaxin | NM_004811 | 1.3 |
| ZNF419 | zinc finger protein 419 | NM_024691 | 1.3 |
| LIME1 | Lck interacting transmembrane adaptor 1 | NM_017806 | 1.3 |
| TOM1 | target of myb1 | NM_005488 | 1.3 |
| SARS | seryl-tRNA synthetase | NM_006513 | 1.3 |
| SNIP1 | Smad nuclear interacting protein 1 | NM_024700 | 1.3 |
| SYT11 | synaptotagmin XI | NM_152280 | 1.3 |
| C16orf58 | chromosome 16 open reading frame 58 | NM_022744 | 1.3 |
| HEY1 | hairy/enhancer-of-split related with YRPW motif 1 | NM_012258 | 1.3 |
| ASB3 | ankyrin repeat and SOCS box-containing 3 | NM_145863 | 1.3 |
| EDF1 | endothelial differentiation-related factor 1 | NM_003792 | 1.3 |
| SLC3A2 | solute carrier family 3, member 2, | NM_001013251 | 1.3 |
| SIL1 | SIL1 homolog, endoplasmic reticulum chaperone | NM_001037633 | 1.3 |
| GALK2 | galactokinase 2 | NM_002044 | 1.3 |
| C17orf70 | chromosome 17 open reading frame 70 | NM_025161 | 1.3 |
| NDEL1 | nudE nuclear distribution gene E homolog-like 1 | NM_030808 | 1.3 |
| ARMET | arginine-rich, mutated in early stage tumors | NM_006010 | 1.3 |
| GSDM1 | gasdermin 1 | NM_178171 | 1.3 |
| JSRP1 | junctional sarcoplasmic reticulum protein 1 | NM_144616 | 1.3 |
| DNAJB2 | DnaJ homolog, subfamily B, member 2 | NM_006736 | 1.3 |
| SSBP2 | single-stranded DNA binding protein 2 | NM_012446 | 1.3 |
| AGPAT4 | 1-acylglycerol-3-phosphate O-acyltransferase 4 | NM_001012734 | 1.3 |
| CXYorf3 | splicing factor, arginine/serine-rich 17A | NM_005088 | 1.3 |
| SCYL1 | SCY1-like 1 | NM_020680 | 1.3 |
| PCNX | pecanex homolog | NM_014982 | 1.3 |
| TAF6L | TAF6-like RNA polymerase II, p300/CBP-associated factor-associated factor, 65kDa | NM_006473 | 1.3 |
| APBB3 | amyloid beta precursor protein-binding, family B, member 3 | NM_006051 | 1.3 |
| SDF2L1 | stromal cell-derived factor 2-like 1 | NM_022044 | 1.3 |
| GABRG2 | gamma-aminobutyric acid A receptor, gamma 2 | NM_198904 | 1.3 |
| ZNF274 | zinc finger protein 274 | NM_016324 | 1.3 |
| C1orf24 | chromosome 1 open reading frame 24 | NM_022083 | 1.3 |
| NANS | N-acetylneuraminic acid synthase | NM_018946 | 1.3 |
| HECTD3 | HECT domain containing 3 | NM_024602 | 1.3 |
| PPM1M | protein phosphatase 1M | NM_144641 | 1.3 |
| KIAA1688 | KIAA1688 protein | NM_025251 | 1.3 |
| KLF11 | Kruppel-like factor 11 | NM_003597 | 1.3 |
| OVGP1 | oviductal glycoprotein 1, 120kDa | NM_002557 | 1.3 |
| ZCCHC8 | zinc finger, CCHC domain containing 8 | NM_017612 | 1.3 |
| BHLHB2 | basic helix-loop-helix domain containing, class B, 2 | NM_003670 | 1.3 |
| C1orf26 | chromosome 1 open reading frame 26 | NM_017673 | 1.3 |
| DNASE2 | deoxyribonuclease II, lysosomal | NM_001375 | 1.3 |
| TUBB3 | tubulin, beta 3 | NM_006086 | 1.3 |
| AARS | alanyl-tRNA synthetase | NM_001605 | 1.3 |
| HSPA5 | heat shock 70kDa protein 5 | NM_005347 | 1.3 |
| MAX | MYC associated factor X | NM_145114 | 1.3 |
| RAB24 | RAB24, member RAS oncogene family | NM_001031677 | 1.3 |
| TXNIP | thioredoxin interacting protein | NM_006472 | 1.2 |
| SLC25A25 | solute carrier family 25, member 25, nuclear gene encoding mitochondrial protein | NM_052901 | 1.2 |
| SLC25A22 | solute carrier family 25, member 22 | NM_024698 | 1.2 |
| CLK3 | CDC-like kinase 3 | NM_003992 | 1.2 |
| SQSTM1 | sequestosome 1 | NM_003900 | 1.2 |
| GMPPA | GDP-mannose pyrophosphorylase A | NM_013335 | 1.2 |
| POGZ | pogo transposable element with ZNF domain | NM_145796 | 1.2 |
| YIF1B | Yip1 interacting factor homolog B, | NM_001031731 | 1.2 |
| ARNTL | aryl hydrocarbon receptor nuclear translocator-like | NM_001030273 | 1.2 |
| CLCN6 | chloride channel 6 | NM_001286 | 1.2 |
| BRSK1 | BR serine/threonine kinase 1 | NM_032430 | 1.2 |
| ZNF331 | zinc finger protein 331 | NM_018555 | 1.2 |
| H1F0 | H1 histone family, member 0 | NM_005318 | 1.2 |
| KLHL22 | kelch-like 22 | NM_032775 | 1.2 |
| LOC168474 | PREDICTED: selenophosphate synthetase pseudogene, misc RNA | XR_000552 | 1.2 |
| RBM21 | terminal uridylyl transferase 1, U6 snRNA-specific | NM_022830 | 1.2 |
| MCEE | methylmalonyl CoA epimerase | NM_032601 | 1.2 |
| FLJ20643 | PIH1 domain containing 1 | NM_017916 | 1.2 |
| PARP8 | poly polymerase family, member 8 | NM_024615 | 1.2 |
| ICA1 | islet cell autoantigen 1, 69kDa | NM_004968 | 1.2 |
| CABC1 | chaperone, ABC1 activity of bc1 complex like | NM_020247 | 1.2 |
| ZNF627 | zinc finger protein 627 | NM_145295 | 1.2 |
| HAX1 | HCLS1 associated protein X-1 | NM_001018837 | 1.2 |
| TMF1 | TATA element modulatory factor 1 | NM_007114 | 1.2 |
| ZNF690 | zinc finger protein 690 | NM_152455 | 1.2 |
| LONRF3 | LON peptidase N-terminal domain and ring finger 3 | NM_024778 | 1.2 |
| AP4B1 | adaptor-related protein complex 4, beta 1 subunit | NM_006594 | 1.2 |
| STC2 | stanniocalcin 2 | NM_003714 | 1.2 |
| ZNF224 | zinc finger protein 224 | NM_013398 | 1.2 |
| LOC51255 | ring finger protein 181 | NM_016494 | 1.2 |
| PSPC1 | paraspeckle component 1 | NM_018282 | 1.2 |
| YY1AP1 | YY1 associated protein 1 | NM_139119 | 1.2 |
| RASSF1 | Ras association domain family 1 | NM_007182 | 1.2 |
| MED8 | mediator of RNA polymerase II transcription, subunit 8 homolog | NM_001001651 | 1.2 |
| YARS | tyrosyl-tRNA synthetase | NM_003680 | 1.2 |
| HMG2L1 | high-mobility group protein 2-like 1 | NM_014250 | 1.2 |
| OSBP | oxysterol binding protein | NM_002556 | 1.2 |
| C20orf111 | chromosome 20 open reading frame 111 | NM_016470 | 1.2 |
| SEC11L3 | SEC11 homolog C | NM_033280 | 1.2 |
| OLIG2 | oligodendrocyte lineage transcription factor 2 | NM_005806 | 1.2 |
| PSMC5 | proteasome 26S subunit, ATPase, 5 | NM_002805 | 1.2 |
| BRF2 | BRF2, subunit of RNA polymerase III transcription initiation factor, BRF1-like | NM_018310 | 1.2 |
| HNRPDL | heterogeneous nuclear ribonucleoprotein D-like | NM_031372 | 1.2 |
| NUDT22 | nudix-type motif 22 | NM_032344 | 1.2 |
| CALR | calreticulin | NM_004343 | 1.2 |
| CRB3 | crumbs homolog 3 | NM_139161 | 1.2 |
| C12orf62 | chromosome 12 open reading frame 62 | NM_032901 | 1.2 |
| STK40 | serine/threonine kinase 40 | NM_032017 | 1.2 |
| ANXA5 | annexin A5 | NM_001154 | 1.2 |
| NCSTN | nicastrin | NM_015331 | 1.2 |
| NME7 | non-metastatic cells 7, protein expressed in | NM_197972 | 1.2 |
| LAT1-3TM | PREDICTED: SLC7A5 pseudogene | XR_000518 | 1.2 |
| FRG1 | FSHD region gene 1 | NM_004477 | 1.2 |
| ZNF621 | zinc finger protein 621 | NM_198484 | 1.2 |
| DCTN2 | dynactin 2 | NM_006400 | 1.2 |
| GSS | glutathione synthetase | NM_000178 | 0.8 |
| TMEM48 | transmembrane protein 48 | NM_018087 | 0.8 |
| HMGB1 | high-mobility group box 1 | NM_002128 | 0.8 |
| DSG2 | desmoglein 2 | NM_001943 | 0.8 |
| DNM3 | dynamin 3 | NM_015569 | 0.8 |
| GEMIN4 | gem associated protein 4 | NM_015721 | 0.8 |
| ASPM | asp-like, microcephaly associated | NM_018136 | 0.8 |
| EFHD2 | EF-hand domain family, member D2 | NM_024329 | 0.8 |
| NIN | ninein | NM_182945 | 0.8 |
| PPAT | phosphoribosyl pyrophosphate amidotransferase | NM_002703 | 0.8 |
| EVA1 | epithelial V-like antigen 1 | NM_144765 | 0.8 |
| C20orf129 | chromosome 20 open reading frame 129 | NM_030919 | 0.8 |
| FLJ40629 | cytoskeleton associated protein 2-like | NM_152515 | 0.8 |
| PLXNA1 | plexin A1 | NM_032242 | 0.8 |
| MTAP | methylthioadenosine phosphorylase | NM_002451 | 0.8 |
| C1orf135 | chromosome 1 open reading frame 135 | NM_024037 | 0.8 |
| LDB2 | LIM domain binding 2 | NM_001290 | 0.8 |
| DARS | aspartyl-tRNA synthetase | NM_001349 | 0.8 |
| STK33 | serine/threonine kinase 33 | NM_030906 | 0.8 |
| KITLG | KIT ligand | NM_000899 | 0.8 |
| LCMT2 | leucine carboxyl methyltransferase 2 | NM_014793 | 0.8 |
| CEP152 | centrosomal protein 152kDa | NM_014985 | 0.8 |
| PARVB | parvin, beta | NM_013327 | 0.8 |
| PLK4 | polo-like kinase 4 | NM_014264 | 0.8 |
| BEX1 | brain expressed, X-linked 1 | NM_018476 | 0.8 |
| MRPS27 | mitochondrial ribosomal protein S27, nuclear gene encoding mitochondrial protein | NM_015084 | 0.8 |
| CDK2 | cyclin-dependent kinase 2 | NM_001798 | 0.8 |
| MGC11257 | hypothetical protein MGC11257 | NM_032350 | 0.8 |
| MGC52057 | LY6/PLAUR domain containing 6 | NM_194317 | 0.8 |
| QKI | quaking homolog, KH domain RNA binding | NM_206853 | 0.8 |
| BCAP31 | B-cell receptor-associated protein 31 | NM_005745 | 0.8 |
| COL9A3 | collagen, type IX, alpha 3 | NM_001853 | 0.8 |
| DLG7 | discs, large homolog 7 | NM_014750 | 0.8 |
| CYYR1 | cysteine/tyrosine-rich 1 | NM_052954 | 0.8 |
| GSTP1 | glutathione S-transferase pi | NM_000852 | 0.8 |
| FAM38A | family with sequence similarity 38, member A | NM_014745 | 0.8 |
| PDSS1 | prenyl diphosphate synthase, subunit 1 | NM_014317 | 0.8 |
| PCDH17 | protocadherin 17 | NM_014459 | 0.8 |
| ACY1L2 | aminoacylase 1-like 2 | NM_001010853 | 0.8 |
| MRPL23 | mitochondrial ribosomal protein L23, nuclear gene encoding mitochondrial protein | NM_021134 | 0.8 |
| CEP55 | centrosomal protein 55kDa | NM_018131 | 0.8 |
| LOC441046 | hypothetical LOC 441046 | NM_001011539 | 0.8 |
| TAP2 | transporter 2, ATP-binding cassette, sub-family B | NM_018833 | 0.8 |
| AKR1B10 | aldo-keto reductase family 1, member B10 | NM_020299 | 0.8 |
| ZNF643 | zinc finger protein 643 | NM_023070 | 0.8 |
| POU4F1 | POU domain, class 4, transcription factor 1 | NM_006237 | 0.8 |
| HNT | neurotrimin | NM_016522 | 0.8 |
| RABGGTA | Rab geranylgeranyltransferase, alpha subunit | NM_004581 | 0.8 |
| LY6E | lymphocyte antigen 6 complex, locus E | NM_002346 | 0.8 |
| FLJ25416 | chromosome 11 open reading frame 82 | NM_145018 | 0.8 |
| HINT2 | histidine triad nucleotide binding protein 2 | NM_032593 | 0.8 |
| AK3 | adenylate kinase 3 | NM_016282 | 0.8 |
| H2AFV | H2A histone family, member V | NM_138635 | 0.8 |
| SMC2 | structural maintenance of chromosomes 2 | NM_001042550 | 0.8 |
| LASS6 | LAG1 homolog, ceramide synthase 6 | NM_203463 | 0.8 |
| TTK | TTK protein kinase | NM_003318 | 0.8 |
| LOC648293 | PREDICTED: similar to RNA-binding motif, single-stranded interacting protein 1 | XM_944019 | 0.8 |
| MMD | monocyte to macrophage differentiation-associated | NM_012329 | 0.8 |
| C18orf54 | chromosome 18 open reading frame 54 | NM_173529 | 0.8 |
| FLJ20105 | excision repair cross-complementing rodent repair deficiency, complementation group 6-like | NM_017669 | 0.8 |
| PGM2 | phosphoglucomutase 2 | NM_018290 | 0.8 |
| AKT1 | v-akt murine thymoma viral oncogene homolog 1 | NM_001014432 | 0.8 |
| RAB7L1 | RAB7, member RAS oncogene family-like 1 | NM_003929 | 0.8 |
| STXBP6 | syntaxin binding protein 6 | NM_014178 | 0.8 |
| PODXL2 | podocalyxin-like 2 | NM_015720 | 0.8 |
| SEC10L1 | SEC10-like 1 | NM_006544 | 0.8 |
| CD59 | CD59 molecule, complement regulatory protein | NM_203329 | 0.8 |
| RANGAP1 | Ran GTPase activating protein 1 | NM_002883 | 0.8 |
| SCML2 | sex comb on midleg-like 2 | NM_006089 | 0.8 |
| DIXDC1 | DIX domain containing 1 | NM_001037954 | 0.8 |
| WDR74 | PREDICTED: WD repeat domain 74 | XM_936269 | 0.8 |
| BUB1 | BUB1 budding uninhibited by benzimidazoles 1 homolog | NM_004336 | 0.8 |
| CHST1 | carbohydrate sulfotransferase 1 | NM_003654 | 0.8 |
| DLNB14 | coiled-coil domain containing 84 | NM_198489 | 0.8 |
| AURKA | aurora kinase A | NM_198434 | 0.8 |
| SMC2L1 | SMC2 structural maintenance of chromosomes 2-like 1 | NM_006444 | 0.8 |
| ATP11C | ATPase, Class VI, type 11C | NM_173694 | 0.8 |
| C17orf69 | chromosome 17 open reading frame 69 | NM_152466 | 0.8 |
| DPM2 | dolichyl-phosphate mannosyltransferase polypeptide 2, regulatory subunit | NM_003863 | 0.8 |
| VGLL3 | vestigial like 3 | NM_016206 | 0.8 |
| TMEFF2 | transmembrane protein with EGF-like and two follistatin-like domains 2 | NM_016192 | 0.8 |
| RAP1A | RAP1A, member of RAS oncogene family | NM_002884 | 0.8 |
| C20orf108 | chromosome 20 open reading frame 108 | NM_080821 | 0.8 |
| COQ10A | coenzyme Q10 homolog A | NM_144576 | 0.8 |
| CPVL | carboxypeptidase, vitellogenic-like | NM_031311 | 0.8 |
| ANLN | anillin, actin binding protein | NM_018685 | 0.8 |
| KNTC1 | kinetochore associated 1 | NM_014708 | 0.8 |
| NDUFS1 | NADH dehydrogenase Fe-S protein 1, 75kDa, nuclear gene encoding mitochondrial protein | NM_005006 | 0.8 |
| NOL3 | nucleolar protein 3 | NM_003946 | 0.8 |
| DTL | denticleless homolog | NM_016448 | 0.8 |
| C15orf29 | chromosome 15 open reading frame 29 | NM_024713 | 0.8 |
| TARDBP | TAR DNA binding protein | NM_007375 | 0.7 |
| TMEM178 | transmembrane protein 178 | NM_152390 | 0.7 |
| SACS | spastic ataxia of Charlevoix-Saguenay | NM_014363 | 0.7 |
| FAM122B | family with sequence similarity 122B | NM_145284 | 0.7 |
| LOC128977 | hypothetical protein LOC128977 | NM_173793 | 0.7 |
| C21orf70 | chromosome 21 open reading frame 70 | NM_058190 | 0.7 |
| SERPINB7 | serpin peptidase inhibitor, clade B, member 7 | NM_003784 | 0.7 |
| DKK2 | dickkopf homolog 2 | NM_014421 | 0.7 |
| MRPL22 | mitochondrial ribosomal protein L22, nuclear gene encoding mitochondrial protein | NM_014180 | 0.7 |
| FHOD1 | formin homology 2 domain containing 1 | NM_013241 | 0.7 |
| ETV3 | ets variant gene 3 | NM_005240 | 0.7 |
| AURKB | aurora kinase B | NM_004217 | 0.7 |
| TMEM64 | transmembrane protein 64 | NM_001008495 | 0.7 |
| FRMD6 | FERM domain containing 6 | NM_152330 | 0.7 |
| HS6ST2 | heparan sulfate 6-O-sulfotransferase 2 | NM_001077188 | 0.7 |
| TNFAIP8L1 | tumor necrosis factor, alpha-induced protein 8-like 1 | NM_152362 | 0.7 |
| C15orf42 | chromosome 15 open reading frame 42 | NM_152259 | 0.7 |
| C8orf72 | chromosome 8 open reading frame 72 | NM_147189 | 0.7 |
| EIF2C2 | eukaryotic translation initiation factor 2C, 2 | NM_012154 | 0.7 |
| PFAS | phosphoribosylformylglycinamidine synthase | NM_012393 | 0.7 |
| RBM12B | RNA binding motif protein 12B | NM_203390 | 0.7 |
| KIF15 | kinesin family member 15 | NM_020242 | 0.7 |
| CRI1 | EP300 interacting inhibitor of differentiation 1 | NM_014335 | 0.7 |
| C15orf41 | chromosome 15 open reading frame 41 | NM_032499 | 0.7 |
| JRK | jerky homolog | NM_003724 | 0.7 |
| FLJ22639 | hypothetical protein FLJ22639 | NM_024796 | 0.7 |
| IMPDH2 | IMP dehydrogenase 2 | NM_000884 | 0.7 |
| SETD1A | SET domain containing 1A | NM_014712 | 0.7 |
| LASS5 | LAG1 homolog, ceramide synthase 5 | NM_147190 | 0.7 |
| FZD4 | frizzled homolog 4 | NM_012193 | 0.7 |
| ZNHIT1 | zinc finger, HIT type 1 | NM_006349 | 0.7 |
| ZNF341 | zinc finger protein 341 | NM_032819 | 0.7 |
| ARL4C | ADP-ribosylation factor-like 4C | NM_005737 | 0.7 |
| MCM3APAS | MCM3 minichromosome maintenance deficient 3 associated protein antisense on chromosome 21 | NR_002776 | 0.7 |
| PUSL1 | pseudouridylate synthase-like 1 | NM_153339 | 0.7 |
| NUDT6 | nudix-type motif 6 | NM_007083 | 0.7 |
| BEX2 | brain expressed X-linked 2 | NM_032621 | 0.7 |
| PTGFRN | prostaglandin F2 receptor negative regulator | NM_020440 | 0.7 |
| LHX2 | LIM homeobox 2 | NM_004789 | 0.7 |
| ALMS1 | Alstrom syndrome 1 | NM_015120 | 0.7 |
| SLC6A15 | solute carrier family 6, member 15 | NM_182767 | 0.7 |
| LOC388503 | similar to Complement C3 precursor | NM_001013640 | 0.7 |
| RNH1 | ribonuclease/angiogenin inhibitor 1 | NM_203385 | 0.7 |
| ELMO3 | engulfment and cell motility 3 | NM_024712 | 0.7 |
| AFAP1L2 | actin filament associated protein 1-like 2 | NM_032550 | 0.7 |
| ME2 | malic enzyme 2, NAD(+)-dependent, mitochondrial, nuclear gene encoding mitochondrial protein | NM_002396 | 0.7 |
| KIAA0195 | KIAA0195 | NM_014738 | 0.7 |
| FLJ22222 | hypothetical protein FLJ22222 | NM_175902 | 0.7 |
| C9orf84 | chromosome 9 open reading frame 84 | NM_173521 | 0.6 |
| FAM102B | family with sequence similarity 102, member B | NM_001010883 | 0.6 |
| DREV1 | DORA reverse strand protein 1 | NM_016025 | 0.6 |
| MKL2 | MKL/myocardin-like 2 | NM_014048 | 0.6 |
| PCGF5 | polycomb group ring finger 5 | NM_032373 | 0.6 |
| TRMT1 | TRM1 tRNA methyltransferase 1 homolog | NM_017722 | 0.6 |
| NMT1 | N-myristoyltransferase 1 | NM_021079 | 0.6 |
| SH3GL1 | SH3-domain GRB2-like 1 | NM_003025 | 0.6 |
| ELF4 | E74-like factor 4 | NM_001421 | 0.6 |
| ALDH1L1 | aldehyde dehydrogenase 1 family, member L1 | NM_012190 | 0.6 |
| C6orf85 | chromosome 6 open reading frame 85 | NM_021945 | 0.6 |
| ICAM3 | intercellular adhesion molecule 3 | NM_002162 | 0.6 |
| PPP3CC | protein phosphatase 3, catalytic subunit, gamma isoform | NM_005605 | 0.6 |
| BHLHB5 | basic helix-loop-helix domain containing, class B, 5 | NM_152414 | 0.6 |
| CALML5 | calmodulin-like 5 | NM_017422 | 0.6 |
| PARL | presenilin associated, rhomboid-like, nuclear gene encoding mitochondrial protein | NM_018622 | 0.6 |
| UHMK1 | U2AF homology motif kinase 1 | NM_175866 | 0.5 |
| PPP1R3D | protein phosphatase 1, regulatory subunit 3D | NM_006242 | 0.5 |
| C12orf44 | chromosome 12 open reading frame 44 | NM_021934 | 0.5 |
| PPAN | peter pan homolog | NM_020230 | 0.5 |
| ZNF143 | zinc finger protein 143 | NM_003442 | 0.5 |
| TKT | transketolase | NM_001064 | 0.4 |
| MDK | midkine | NM_001012334 | 0.4 |
| PSARL | presenilin associated, rhomboid-like | NM_018622 | 0.3 |
| TMEM11 | transmembrane protein 11 | NM_003876 | 0.3 |
| SNX15 | sorting nexin 15 | NM_013306 | 0.3 |
| TCTA | T-cell leukemia translocation altered gene | NM_022171 | 0.3 |
| C1orf115 | chromosome 1 open reading frame 115 | NM_024709 | 0.3 |
| C14orf94 | chromosome 14 open reading frame 94 | NM_017815 | 0.3 |
| The gene expression profile of NSCLC line NCI-H520 transiently transfected cells (4 replicas) was analyzed using the Human-8 v3 Expression BeadChips (Illumina Inc., San Diego, CA). The data set was normalized using a cubic spline algorithm, with BeadStudio Version 3 software. A P-value<0.05 was set as a cutoff to filter reliably detected genes. | | | |
