# Supplementary material for: MFSD2A is a novel lung tumor suppressor gene modulating cell cycle and matrix attachment
Source: Mol Cancer. 2010 Mar 17;9:62. doi: 10.1186/1476-4598-9-62 (PMC2846890; doi:10.1186/1476-4598-9-62)
Supplement: Additional file 3 — List of genes differentially expressed between lung cancer cell lines by MFSD2A mRNA levels. The gene expression profile of 47 NSCLC cell lines was analyzed on the Affymetrix GeneChips HG-U133A and HG-U133B. Arrays from both types were pooled and normalized to their 100 common control genes. Groups of samples were compared by calculating log2 ratios for each gene. A T-test P-value < 0.005 was set as a cutoff to select differentially expressed genes. [file 1476-4598-9-62-S3.DOC]

| Additional file 3. List of genes differentially expressed between lung cancer cell lines showing the highest and lowest MFSD2A mRNA levels (P<0.005, fold change≥4) and sorted by fold change ^a^. | | | |
| --- | --- | --- | --- |
| Gene symbol | Gene name | Gene accession # | Fold change (log_2_ ratio, high/low MFSD2A) |
| INSM1 | Insulinoma-associated 1 | NM_002196 | 6.63 |
| ASCL1 | Achaete-scute complex homolog 1 (Drosophila) | NM_004316 | 6.28 |
| MIAT | Myocardial infarction associated transcript (non-protein coding) | CR620293 | 5.90 |
| SYT4 | Synaptotagmin IV | NM_020783 | 5.42 |
| HES6 | Hairy and enhancer of split 6 (Drosophila) | NM_018645 | 5.09 |
| NOL4 | Nucleolar protein 4 | NM_003787 | 4.89 |
| TMSL8 | Thymosin-like 8 | NM_021992 | 4.53 |
| SCN3A | Sodium channel, voltage-gated, type III, alpha subunit | NM_006922 | 4.45 |
| SOX11 | SRY (sex determining region Y)-box 11 | NM_003108 | 4.39 |
| ST18 | Suppression of tumorigenicity 18 (breast carcinoma) (zinc finger protein) | NM_014682 | 4.22 |
| PROM1 | Prominin 1 | NM_006017 | 4.05 |
| MYCL1 | V-myc myelocytomatosis viral oncogene homolog 1, lung carcinoma derived (avian) | NM_001033082 | 4.04 |
| BEX1 | Brain expressed, X-linked 1 | NM_018476 | 4.04 |
| ELAVL3 | ELAV (embryonic lethal, abnormal vision, Drosophila)-like 3 (Hu antigen C) | NM_032281 | 3.94 |
| DACH1 | Dachshund homolog 1 (Drosophila) | NM_080760 | 3.87 |
| DLL1 | Delta-like 1 (Drosophila) | NM_005618 | 3.79 |
| PTPRZ1 | Protein tyrosine phosphatase, receptor-type, Z polypeptide 1 | NM_002851 | 3.76 |
| LOC253012 | Hypothetical protein LOC253012 | NM_001039372 | 3.65 |
| SP8 | Sp8 transcription factor | NM_198956 | 3.64 |
| NHLH2 | Nescient helix loop helix 2 | NM_005599 | 3.60 |
| PGBD5 | PiggyBac transposable element derived 5 | NM_024554 | 3.58 |
| ELAVL4 | ELAV (embryonic lethal, abnormal vision, Drosophila)-like 4 (Hu antigen D) | NM_021952 | 3.52 |
| FXYD6 | FXYD domain containing ion transport regulator 6 | NM_022003 | 3.51 |
| DPYSL5 | Dihydropyrimidinase-like 5 | AK022795 | 3.44 |
| SEZ6 | Seizure related 6 homolog (mouse) | NM_178860 | 3.42 |
| PCSK1 | Proprotein convertase subtilisin/kexin type 1 | NM_000439 | 3.40 |
| CHGA | Chromogranin A (parathyroid secretory protein 1) | NM_001275 | 3.34 |
| CRMP1 | Collapsin response mediator protein 1 | NM_001313 | 3.34 |
| C7orf41 | Chromosome 7 open reading frame 41 | NM_152793 | 3.32 |
| MFSD2A | Major facilitator superfamily domain containing 2 | NM_032793 | 3.31 |
| TCEAL2 | Transcription elongation factor A (SII)-like 2 | NM_080390 | 3.31 |
| NPNT | Nephronectin | NM_001033047 | 3.30 |
| NCAM1 | Neural cell adhesion molecule 1 | BC014205 | 3.27 |
| ISL1 | ISL1 transcription factor, LIM/homeodomain, (islet-1) | NM_002202 | 3.26 |
| RUNX1T1 | Runt-related transcription factor 1; translocated to, 1 (cyclin D-related) | NM_004349 | 3.23 |
| CXCR4 | Chemokine (C-X-C motif) receptor 4 | NM_001008540 | 3.20 |
| SOX2 | SRY (sex determining region Y)-box 2 | NM_003106 | 3.18 |
| IGFBPL1 | Insulin-like growth factor binding protein-like 1 | AK055716 | 3.18 |
| PPM1E | Protein phosphatase 1E (PP2C domain containing) | NM_014906 | 3.15 |
| POU4F1 | POU domain, class 4, transcription factor 1 | NM_006237 | 3.13 |
| TAGLN3 | Transgelin 3 | NM_013259 | 3.13 |
| NRXN1 | Neurexin 1 | AB011150 | 3.12 |
| TOX | Thymocyte selection-associated high mobility group box | NM_014729 | 3.10 |
| SETBP1 | SET binding protein 1 | NM_015559 | 3.07 |
| KIT | V-kit Hardy-Zuckerman 4 feline sarcoma viral oncogene homolog | NM_000222 | 3.02 |
| CNTNAP2 | Contactin associated protein-like 2 | NM_014141 | 2.96 |
| ABAT | 4-aminobutyrate aminotransferase | NM_020686 | 2.92 |
| AUTS2 | Autism susceptibility candidate 2 | NM_015570 | 2.91 |
| RTN1 | Reticulon 1 | NM_206857 | 2.89 |
| KIAA1909 | KIAA1909 protein | NM_052909 | 2.86 |
| GAD1 | Glutamate decarboxylase 1 (brain, 67kDa) | NM_000817 | 2.83 |
| CACNA1A | Calcium channel, voltage-dependent, P/Q type, alpha 1A subunit | BC028611 | 2.82 |
| C19orf30 | Chromosome 19 open reading frame 30 | NM_174947 | 2.81 |
| DNALI1 | Dynein, axonemal, light intermediate chain 1 | NM_003462 | 2.81 |
| GABRB3 | Gamma-aminobutyric acid (GABA) A receptor, beta 3 | NM_021912 | 2.74 |
| PCP4L1 | Purkinje cell protein 4 like 1 | XM_929467 | 2.71 |
| PDZRN3 | PDZ domain containing RING finger 3 | NM_015009 | 2.69 |
| SBK1 | SH3-binding domain kinase 1 | NM_001024401 | 2.68 |
| TUBB2B | Tubulin, beta 2B | NM_178012 | 2.68 |
| CADM1 | Cell adhesion molecule 1 | AK172730 | 2.67 |
| PPP1R1B | Protein phosphatase 1, regulatory (inhibitor) subunit 1B (dopamine and cAMP regulated phosphoprotein, DARPP-32) | NM_181505 | 2.60 |
| SH3GL2 | SH3-domain GRB2-like 2 | NM_003026 | 2.60 |
| FNDC5 | Fibronectin type III domain containing 5 | NM_153756 | 2.59 |
| CD200 | CD200 molecule | NM_001004196 | 2.58 |
| RELN | Reelin | NM_005045 | 2.58 |
| CDK5R1 | Cyclin-dependent kinase 5, regulatory subunit 1 (p35) | NM_003885 | 2.56 |
| MTSS1 | Metastasis suppressor 1 | NM_014751 | 2.56 |
| BCL2 | B-cell CLL/lymphoma 2 | NM_000633 | 2.55 |
| NELL2 | NEL-like 2 (chicken) | NM_006159 | 2.55 |
| ONECUT2 | One cut domain, family member 2 | CR590917 | 2.54 |
| MAP2 | Microtubule-associated protein 2 | NM_001039538 | 2.50 |
| KIAA0367 | KIAA0367 | NM_015225 | 2.49 |
| TMEM108 | Transmembrane protein 108 | NM_023943 | 2.47 |
| CA8 | Carbonic anhydrase VIII | NM_004056 | 2.47 |
| TCF4 | Transcription factor 4 | AK095066 | 2.45 |
| CAMK2B | Calcium/calmodulin-dependent protein kinase (CaM kinase) II beta | NM_172084 | 2.45 |
| SV2A | Synaptic vesicle glycoprotein 2A | NM_014849 | 2.44 |
| NFASC | Neurofascin homolog (chicken) | NM_015090 | 2.42 |
| DKFZP761M1511 | Hypothetical protein DKFZP761M1511 | AK026748 | 2.39 |
| SEC11C | SEC11 homolog C (S. cerevisiae) | NM_033280 | 2.39 |
| HOXD10 | Homeobox D10 | NM_002148 | 2.39 |
| ABCA5 | ATP-binding cassette, sub-family A (ABC1), member 5 | NM_172232 | 2.38 |
| GPRC5B | G protein-coupled receptor, family C, group 5, member B | NM_016235 | 2.37 |
| BRUNOL4 | Bruno-like 4, RNA binding protein (Drosophila) | NM_020180 | 2.34 |
| DKFZP761N09121 | Hypothetical protein DKFZp761N09121 | AF038190 | 2.31 |
| TMEM46 | Transmembrane protein 46 | NM_001007538 | 2.31 |
| NRCAM | Neuronal cell adhesion molecule | NM_005010 | 2.30 |
| KCNMB2 | Potassium large conductance calcium-activated channel, subfamily M, beta member 2 | NM_181361 | 2.30 |
| MAGED4 | Melanoma antigen family D, 4 | NM_177537 | 2.29 |
| ZFP3 | Zinc finger protein 3 homolog (mouse) | NM_153018 | 2.27 |
| FAM105A | Family with sequence similarity 105, member A | NM_019018 | 2.24 |
| SDK2 | Sidekick homolog 2 (chicken) | BC066363 | 2.24 |
| SNAP25 | Synaptosomal-associated protein, 25kDa | NM_003081 | 2.21 |
| TRIM9 | Tripartite motif-containing 9 | NM_015163 | 2.20 |
| C6orf60 | Chromosome 6 open reading frame 60 | NM_024581 | 2.19 |
| NRSN1 | Neurensin 1 | NM_080723 | 2.18 |
| TRIT1 | TRNA isopentenyltransferase 1 | NM_017646 | 2.17 |
| REC8 | REC8 homolog (yeast) | NM_005132 | 2.17 |
| ID4 | Inhibitor of DNA binding 4, dominant negative helix-loop-helix protein | AJ420553 | 2.16 |
| PRRT2 | Proline-rich transmembrane protein 2 | NM_145239 | 2.16 |
| ELAVL1 | ELAV (embryonic lethal, abnormal vision, Drosophila)-like 1 (Hu antigen R) | NM_001419 | 2.15 |
| AMACR | Alpha-methylacyl-CoA racemase | BC009471 | 2.15 |
| RIMS3 | Regulating synaptic membrane exocytosis 3 | NM_014747 | 2.14 |
| TOX3 | TOX high mobility group box family member 3 | U80736 | 2.14 |
| APCDD1 | Adenomatosis polyposis coli down-regulated 1 | NM_153000 | 2.13 |
| PSIP1 | PC4 and SFRS1 interacting protein 1 | NM_033222 | 2.13 |
| RAB3IP | RAB3A interacting protein (rabin3) | NM_001024647 | 2.13 |
| ASXL3 | Additional sex combs like 3 (Drosophila) | XM_290811 | 2.13 |
| SNX26 | Sorting nexin 26 | NM_052948 | 2.12 |
| SOX2OT | SOX2 overlapping transcript (non-coding RNA) | AL157425 | 2.11 |
| FAM84A | Family with sequence similarity 84, member A | AL137343 | 2.10 |
| GDAP1 | Ganglioside-induced differentiation-associated protein 1 | NM_018972 | 2.09 |
| LMO2 | LIM domain only 2 (rhombotin-like 1) | NM_005574 | 2.08 |
| FSD1L | Fibronectin type III and SPRY domain containing 1-like | CR936789 | 2.06 |
| DPYSL3 | Dihydropyrimidinase-like 3 | NM_001387 | 2.06 |
| TMEM163 | Transmembrane protein 163 | NM_030923 | 2.06 |
| SYT11 | Synaptotagmin XI | NM_152280 | 2.05 |
| TUBB2A | Tubulin, beta 2A | NM_001069 | 2.05 |
| CHD7 | Chromodomain helicase DNA binding protein 7 | NM_017780 | 2.04 |
| D4S234E | DNA segment on chromosome 4 (unique) 234 expressed sequence | NM_014392 | 2.03 |
| PCLO | Piccolo (presynaptic cytomatrix protein) | AB011131 | 2.03 |
| CADPS2 | Ca2+-dependent activator protein for secretion 2 | NM_017954 | 2.02 |
| ARMCX5 | Armadillo repeat containing, X-linked 5 | NM_014710 | 2.00 |
| BDNF | Brain-derived neurotrophic factor | NM_170735 | -2.00 |
| NT5E | 5'-nucleotidase, ecto (CD73) | NM_002526 | -2.00 |
| GLRX | Glutaredoxin (thioltransferase) | NM_002064 | -2.01 |
| ANXA1 | Annexin A1 | NM_000700 | -2.01 |
| GPR64 | G protein-coupled receptor 64 | NM_005756 | -2.03 |
| AHNAK2 | AHNAK nucleoprotein 2 | XM_290629 | -2.03 |
| FLNC | Filamin C, gamma (actin binding protein 280) | NM_001458 | -2.03 |
| VEGFC | Vascular endothelial growth factor C | NM_005429 | -2.04 |
| RRAS | Related RAS viral (r-ras) oncogene homolog | NM_006270 | -2.04 |
| SMAD3 | SMAD family member 3 | NM_005902 | -2.05 |
| PRICKLE1 | Prickle homolog 1 (Drosophila) | AK122881 | -2.07 |
| NPAS2 | Neuronal PAS domain protein 2 | NM_002518 | -2.07 |
| SRXN1 | Sulfiredoxin 1 homolog (S. cerevisiae) | NM_080725 | -2.08 |
| ME1 | Malic enzyme 1, NADP(+)-dependent, cytosolic | CR591849 | -2.08 |
| KRT86 | Keratin 86 | NM_002284 | -2.08 |
| GABRE | Gamma-aminobutyric acid (GABA) A receptor, epsilon | NM_004961 | -2.09 |
| ITGB4 | Integrin, beta 4 | NM_000213 | -2.11 |
| SMAD6 | SMAD family member 6 | NM_005585 | -2.12 |
| F2RL1 | Coagulation factor II (thrombin) receptor-like 1 | NM_005242 | -2.13 |
| CD97 | CD97 molecule | NM_001784 | -2.16 |
| PDLIM5 | PDZ and LIM domain 5 | NM_006457 | -2.16 |
| LATS2 | LATS, large tumor suppressor, homolog 2 (Drosophila) | BC071572 | -2.16 |
| CRIM1 | Cysteine rich transmembrane BMP regulator 1 (chordin-like) | NM_016441 | -2.20 |
| EMP1 | Epithelial membrane protein 1 | NM_001423 | -2.21 |
| PRSS23 | Protease, serine, 23 | NM_007173 | -2.24 |
| PCDH7 | Protocadherin 7 | NM_002589 | -2.24 |
| WWTR1 | WW domain containing transcription regulator 1 | AL833852 | -2.24 |
| CYR61 | Cysteine-rich, angiogenic inducer, 61 | NM_001554 | -2.26 |
| SLC16A5 | Solute carrier family 16, member 5 (monocarboxylic acid transporter 6) | NM_004695 | -2.26 |
| KIRREL | Kin of IRRE like (Drosophila) | AK090554 | -2.29 |
| GNG11 | Guanine nucleotide binding protein (G protein), gamma 11 | NM_004126 | -2.30 |
| COL6A1 | Collagen, type VI, alpha 1 | NM_001848 | -2.30 |
| TNFRSF10B | Tumor necrosis factor receptor superfamily, member 10b | NM_147187 | -2.32 |
| CCDC80 | Coiled-coil domain containing 80 | NM_199512 | -2.33 |
| C19orf33 | Chromosome 19 open reading frame 33 | NM_033520 | -2.36 |
| TGFB2 | Transforming growth factor, beta 2 | M19154 | -2.36 |
| FER1L3 | Fer-1-like 3, myoferlin (C. elegans) | NM_133337 | -2.42 |
| PRKCDBP | Protein kinase C, delta binding protein | NM_145040 | -2.43 |
| SERPINH1 | Serpin peptidase inhibitor, clade H (heat shock protein 47), member 1, (collagen binding protein 1) | NM_001235 | -2.45 |
| EMP3 | Epithelial membrane protein 3 | NM_001425 | -2.46 |
| MAG1 | Lung cancer metastasis-associated protein | NM_032717 | -2.49 |
| TGFBI | Transforming growth factor, beta-induced, 68kDa | NM_000358 | -2.62 |
| TPM2 | Tropomyosin 2 (beta) | NM_003289 | -2.67 |
| KIAA1913 | KIAA1913 | NM_052913 | -2.69 |
| AHNAK | AHNAK nucleoprotein | NM_001620 | -2.70 |
| SERPINE2 | Serpin peptidase inhibitor, clade E (nexin, plasminogen activator inhibitor type 1), member 2 | NM_006216 | -2.70 |
| OAS1 | 2',5'-oligoadenylate synthetase 1, 40/46kDa | NM_002534 | -2.71 |
| RASSF8 | Ras association (RalGDS/AF-6) domain family 8 | AY665468 | -2.73 |
| NNMT | Nicotinamide N-methyltransferase | NM_006169 | -2.74 |
| CYBRD1 | Cytochrome b reductase 1 | NM_024843 | -2.76 |
| ALDH3B1 | Aldehyde dehydrogenase 3 family, member B1 | NM_000694 | -2.79 |
| PPARG | Peroxisome proliferator-activated receptor gamma | NM_015869 | -2.79 |
| PTRF | Polymerase I and transcript release factor | NM_012232 | -2.81 |
| PROCR | Protein C receptor, endothelial (EPCR) | NM_006404 | -2.83 |
| LOC399959 | Hypothetical gene supported by BX647608 | BC040599 | -2.83 |
| C6orf176 | Chromosome 6 open reading frame 176 | BC039614 | -2.83 |
| TM4SF1 | Transmembrane 4 L six family member 1 | NM_014220 | -2.85 |
| THBS1 | Thrombospondin 1 | NM_003246 | -2.86 |
| ABI3BP | ABI gene family, member 3 (NESH) binding protein | NM_015429 | -2.86 |
| ARL14 | ADP-ribosylation factor-like 14 | NM_025047 | -2.90 |
| NQO1 | NAD(P)H dehydrogenase, quinone 1 | NM_001025434 | -3.01 |
| EREG | Epiregulin | NM_001432 | -3.06 |
| HKDC1 | Hexokinase domain containing 1 | NM_025130 | -3.14 |
| CAV2 | Caveolin 2 | NM_198212 | -3.17 |
| FOSL1 | FOS-like antigen 1 | NM_005438 | -3.17 |
| IGFBP7 | Insulin-like growth factor binding protein 7 | NM_001553 | -3.18 |
| VIM | Vimentin | NM_003380 | -3.21 |
| EDIL3 | EGF-like repeats and discoidin I-like domains 3 | BX648583 | -3.21 |
| ALPK2 | Alpha-kinase 2 | NM_052947 | -3.23 |
| LAMA1 | Laminin, alpha 1 | NM_005559 | -3.33 |
| IL18 | Interleukin 18 (interferon-gamma-inducing factor) | NM_001562 | -3.41 |
| SDPR | Serum deprivation response (phosphatidylserine binding protein) | NM_004657 | -3.45 |
| LGALS1 | Lectin, galactoside-binding, soluble, 1 (galectin 1) | NM_002305 | -3.52 |
| AXL | AXL receptor tyrosine kinase | NM_021913 | -3.53 |
| DKK1 | Dickkopf homolog 1 (Xenopus laevis) | NM_012242 | -3.65 |
| S100A4 | S100 calcium binding protein A4 | NM_002961 | -4.57 |
| CAV1 | Caveolin 1, caveolae protein, 22kDa | NM_001753 | -5.30 |
| ^a^ Cell lines with low MFSD2A mRNA levels: Calu-1, Calu-6, H1299, H1355, H1437, H157, H1819, H23, H2347, H2882, H2887, H460, HCC15, HCC2279, HCC461, HCC515, HCC78. Cell lines with high MFSD2A mRNA levels: H1963, H209, H2171, H2195, H2227, H3255, H889, HCC1195, HCC1833, HCC2935, HCC33, HCC44. The gene expression profile of 47 NSCLC cell lines was analyzed on the Affymetrix GeneChips HG-U133A and HG-U133B. Arrays from both types were pooled and normalized to their 100 common control genes. Groups of samples were compared by calculating log_2_ ratios for each gene. A T-test P-value<0.005 was set as a cutoff to select differentially expressed genes. | | | |
